# Obesity-associated genetic variants in young Asian Indians with the metabolic syndrome and myocardial infarction

**DOI:** 10.5830/CVJA-2010-036

**Published:** 2011-02

**Authors:** Naresh Ranjith, Rosemary J Pegoraro, Rebecca Shanmugam

**Affiliations:** Departments of Medicine, Coronary Care Unit, RK Khan Hospital, Chemical Pathology, Nelson R Mandela School of Medicine, University of KwaZulu-Natal, and Biostatistics Unit, Medical Research Council of South Africa; Departments of Medicine, Coronary Care Unit, RK Khan Hospital, Chemical Pathology, Nelson R Mandela School of Medicine, University of KwaZulu-Natal, and Biostatistics Unit, Medical Research Council of South Africa; Departments of Medicine, Coronary Care Unit, RK Khan Hospital, Chemical Pathology, Nelson R Mandela School of Medicine, University of KwaZulu-Natal, and Biostatistics Unit, Medical Research Council of South Africa

**Keywords:** obesity, metabolic syndrome, myocardial infarction, genetic polymorphisms

## Abstract

**Objective:**

Associations between obesity-related polymorphisms and the metabolic syndrome in 485 young (≤ 45 years) Asian Indian patients with acute myocardial infarction (AMI), and 300 matched controls were assessed.

**Methods:**

Genetic variants included the adiponectin 45T→G and 276G→T, LEPR K109R and Q223R, MC4R-associated C→T and FTO A→T polymorphisms.

**Results:**

The metabolic syndrome, as defined by NCEP ATP III and IDF criteria, was diagnosed in 61 and 60% of patients, respectively. No relationship was found between the obesity-associated polymorphisms and the metabolic syndrome, or between AMI patients and controls. The MC4R-associated TT genotype occurred more frequently in patients with lower triglyceride levels (*p* = 0.024), while the adiponectin 45 TT genotype occurred more commonly in patients with normal fasting glucose levels (*p* = 0.004). The LEPR Q223R TT genotype was associated with low high-density lipoprotein (HDL) cholesterol levels (*p* = 0.003).

**Conclusion:**

The metabolic syndrome occurs commonly in young Asian Indian patients with AMI. No relationship was found between any obesity-associated polymorphism and the metabolic syndrome. Particular genotypes may exert protective or disadvantageous effects on individual components of the metabolic syndrome.

## Summary

Obesity is recognised as a serious chronic disease, the prevalence of which is increasing worldwide. Several studies have demonstrated a strong association between obesity and insulin resistance. This relationship frequently leads to diabetes mellitus, hypertension, dyslipidaemia and vascular inflammation, all of which promote the development of atherosclerotic cardiovascular disease.^1^-^3^ The co-occurrence of these metabolic risk factors has given rise to the metabolic syndrome.

Although there are several definitions for the metabolic syndrome, the National Cholesterol Education Program Adult Treatment Panel III (NCEP ATP III)^4^ and the International Diabetes Federation (IDF)^5^ definitions are the most widely used. Fundamental to the syndrome is the close interaction between abdominal fat patterning, total body adiposity, and insulin resistance. Both definitions include central obesity as one of the criteria, but with different waist circumference cut-offs. The IDF definition of the metabolic syndrome, in fact, proposes central obesity as an essential element, with specific waist circumference thresholds set for different ethnic groups. In addition, the metabolic syndrome is reportedly present in 60% of individuals who are obese,^6^ while an increased waist circumference, taken in isolation, identifies up to 40% of individuals who will develop the syndrome within five years.^7^

Obesity is believed to be a heritable trait. However, the genes that contribute to the less severe but more common forms of obesity have been difficult to identify. Several studies have demonstrated a possible role for the adiponectin gene in obesity, insulin resistance and type 2 diabetes.^8^-^11^ Adiponectin, which is an adipocyte-derived cytokine, has been shown to be down regulated in obesity, particularly in those individuals with visceral obesity, while adiponectin levels correlate inversely with insulin resistance.^12^-^14^ Furthermore, preliminary data suggest that high adiponectin concentrations are associated with a favourable cardiovascular outcome.^15^-^16^ It has been estimated that between 30 and 70% of the variability in adiponectin levels is determined by genetic factors.^17^ Although several polymorphisms in the adiponectin gene have been described, data linking these variants to obesity and related conditions are inconclusive.

Leptin is another important protein associated with obesity, and functions by inhibiting food intake and stimulating energy expenditure.^18^ Leptin levels are regulated by various proteins, one of which is the leptin receptor (LEPR). Several polymorphisms are found in the LEPR gene but earlier studies, which examined potential associations between LEPR gene polymorphisms and obesity, failed to report conclusive results.^19^,^20^ Nevertheless, both the adiponectin and the LEPR genes remain potential candidates in the aetiology of obesity and coronary heart disease (CHD).

Other genes have also been associated with obesity in the general population, most notably the melanocortin-4-receptor (MC4R) and the fat mass and obesity-associated (FTO) genes. Activation of the MC4R gene reduces body fat stores by decreasing food intake and increasing energy expenditure. Functional mutations in the MC4R gene are reportedly associated with hyperphagia, early-onset obesity and hyperinsulinaemia, while polymorphisms near the MC4R gene are linked to increased body mass index (BMI) and abdominal girth.^21^,^22^ Variants in the FTO gene have been associated with obesity in a genome-wide study,^23^ and have been shown to predispose to diabetes through an effect on BMI.^24^

In the present study, we examined single nucleotide polymorphisms (SNPs) in the adiponectin, LEPR, MC4R and FTO genes in association with obesity in a cohort of young South African Asian Indian patients with acute myocardial infarction (AMI), with and without the metabolic syndrome. This study group is of particular interest due to the high incidence of both premature CHD and the metabolic syndrome in the South African Indian population.^25^ The genetic variants selected for study included the adiponectin 45T→G (rs2241766) and 276G→T (rs1501299), the LEPR K109R (rs1173100) and Q223R (rs1173101), the MC4Rassociated C→T (rs17882313) and the FTO A→T (rs9939609) polymorphisms. We also compared the frequencies of these polymorphisms in the AMI subjects with the frequencies found in a control group of people free of CHD, to assess the potential of these polymorphisms as risk factors for CHD.

## Methods

A total of 485 Asian Indian subjects presenting with AMI was studied. The study population and demographic profiles have been described in detail previously.^25^ Briefly, subjects eligible for inclusion were men and women aged 45 years or younger, who were admitted with a diagnosis of AMI based on the Joint European Society of Cardiology/American College of Cardiology Committee definition.^26^ Both the NCEP ATP III and the IDF definitions were used to assess the prevalence of the metabolic syndrome.

The investigation conforms to the principles outlined in the Declaration of Helsinki. Informed consent was obtained from all individuals in the study and approval was granted by the Ethics Committee of the Faculty of Health Sciences, Nelson R Mandela School of Medicine, University of KwaZulu-Natal.

Blood samples were collected from all AMI patients within 48 hours of admission after an overnight fast. Total cholesterol, triglycerides, high-density lipoprotein (HDL) cholesterol and glucose levels were determined using standard enzymatic methods on a Beckman UniCel DxC800 auto analyser.

Anthropometric measurements, including BMI and waist circumference, were used to define obesity. The BMI was calculated as weight (kg) divided by height^2^ (m) according to World Health Organisation guidelines.^27^ A BMI ≥ 30 kg/m^2^ was used as a cut-off to indicate obesity. Waist circumference, which is considered the most practical way to assess central obesity, was measured midway between the lowest rib and the iliac crest on standing subjects, using a soft tape measure. The central obesity threshold limits proposed by both the NCEP ATP III (males > 102 cm, females > 88 cm) and IDF (males ≥ 90 cm, females ≥ 80 cm) were used to define the metabolic syndrome.

The control group comprised 300 healthy age-matched Asian Indian subjects drawn from the same community as the patients. None of these subjects suffered from cardiovascular disease or had any associated clinical risk factors. All were non-smokers and none was obese.

Blood for DNA analysis was collected from both AMI patients and control subjects in ethylenediaminetetra-acetic acid (EDTA) tubes, and stored at –20°C until DNA isolation by standard techniques. Genotyping of all six SNPs was performed by TaqMan SNP allelic discrimination pre-designed assays (rs2241766: catalogue no C 26426077_10; rs1501299: catalogue no C 7497299_10; rs1173100: catalogue no C 7586955_10; rs1173101: catalogue no C 7586956_10, rs17882313: catalogue no C 32667060_10; rs9939609: catalogue no C 30090620_10) using an ABI 7500 thermal cycler (Applied Biosystems). All results were automatically called. Approximately 10% of all samples were genotyped on more than one occasion and showed 100% concordance.

## Statistical analysis

Data were analysed using the STATA, version 11 (StataCorp LP, TX). The Pearson chi-squared or the Fisher exact test, when appropriate, was used to test associations between independent, categorical exposures and outcomes. Confidence intervals were constructed by use of odds ratios, with all confidence intervals assessed at the 95% level of confidence. Where values were of a continuous nature, the *t*-test was used to assess mean values between groups. Differences were considered statistically significant when p < 0.05.

## Results

The biochemical and clinical characteristics of male and female subjects are shown in Table 1. The study population comprised 485 patients, 86% of whom were males. Compared with males, females had significantly higher mean baseline glucose (10.9 ± 5.37 vs 8.44 ± 4.36 mmol/l, *p* = 0.005) and HDL cholesterol levels (1.14 ± 0.35 vs 0.95 ± 0.28 mmol/l, *p* ≤ 0.0001). Triglyceride levels did not differ between genders. The metabolic syndrome as defined by the NCEP ATP III criteria was diagnosed in 61% of patients [males (59%), females (76%)], and in 60% of patients [males (58%), females (73%)] according to the IDF criteria. Significantly more females were found to have the metabolic syndrome as defined by NCEP ATP III and IDF criteria (*p* = 0.007 and 0.02, respectively).

**Table 1.** Baseline Biochemical And Clinical Characteristics Of Patients According To Gender

*Variable*	*Males, n = 419 (86%)*	*Females, n = 66 (14%)*	*Total 485*	p*-value*
Blood glucose (mmol/l)*	8.44 ± 4.36	10.9 ± 5.37	8.67 ± 4.54	**0.005**
Blood pressure (mmHg)*				
Systolic	129 ± 22	128 ± 22	129 ± 22	0.71
Diastolic	81 ± 16	78 ± 13	80 ± 16	0.26
HDL cholesterol (mmol/l)*	0.95 ± 0.28	1.14 ± 0.35	0.98 ± 0.29	**< 0.0001**
Triglycerides (mmol/l)*	2.57 ± 1.84	2.36 ± 1.67	2.54 ± 1.81	0.39
Abdominal circumference (cm)*	96.08 ± 12.87	96.88 ± 11.48	96.18 ± 12.69	0.66
Metabolic syndrome (NCEP ATP III)**	245 (59%)	50 (76%)	295 (61%)	**0.007**
Metabolic syndrome (IDF)**	242 (58%)	48 (73%)	290 (60%)	**0.02**
BMI (≥ 30 kg/m^2^)**	72 (17%)	21 (32%)	93 (19%)	**0.002**

*Values expressed as mean ± standard deviation; **number of individuals (% total). Figures in bold show significance at the 5% level. NCEP ATP III: National Cholesterol Education Program ATP III, IDF: International Diabetes Federation, HDL: high-density lipoprotein, BMI: body mass index.

With respect to BMI measurements, 19% of all subjects were classified as obese (BMI ≥ 30 kg/m^2^), with proportionally more females (32%) than males (17%, *p* = 0.002). Forty-five per cent of patients had an increased waist circumference (visceral obesity) based on the NCEP ATP III definition of the syndrome (mean abdominal girth 108.73 ± 10.81 cm). In the 290 patients with the metabolic syndrome according to the IDF criteria, the mean abdominal girth was expectedly lower (102.50 ± 10.32 cm), because of the lower limits set for waist circumference measurements in the Asian population.

The genotype and allele frequency distributions of the adiponectin, LEPR, MC4R and FTO gene polymorphisms in relation to the metabolic syndrome as defined by the NCEP ATP III and IDF criteria are shown in Tables 2 and 3, respectively. The frequencies of all polymorphisms were in Hardy-Weinberg equilibrium. No significant relationship was found between any of the obesity-associated polymorphisms and the metabolic syndrome, irrespective of the definition used, nor were there any differences in polymorphic frequencies with respect to gender, or between patients and controls for both definitions of the syndrome (data not shown).

**Table 2.** Genotype And allele Frequencies Of Obesity-Associated Polymorphisms In Patients With And Without The Metabolic Syndrome As Determined By The NCEP ATP III Definition

*Genotype/allelotype*	*NCEP (yes) n (%)*	*NCEP (no) n (%)*	*OR (95% CI)*	p*-value*
Adiponectin 45 T→G				
TT	208 (71)	134 (71)	1.00 (0.66–1.53)	1.000
TG	81 (27)	50 (26)	0.94 (0.61–1.45)	0.834
GG	6 (2)	6 (3)	1.57 (0.41–5.96)	0.552
T allele	497 (84)	318 (84)	0.96 (0.67–1.39)	0.858
G allele	93 (16)	62 (16)	1.04 (0.72–1.50)	0.858
Adiponectin 276 G→T				
GG	181 (61)	113 (59)	0.92 (0.63–1.37)	0.704
GT	99 (34)	65 (34)	1.03 (0.69–1.54)	0.922
TT	15 (5)	12 (6)	1.26 (0.53–2.95)	0.551
G allele	461 (78)	291 (77)	0.92 (0.67–1.26)	0.582
T allele	129 (22)	89 (23)	1.09 (0.79–1.50)	0.582
LEPR K109R				
AA	220 (75)	130 (68)	0.74 (0.48–1.13)	0.147
AG	69 (23)	56 (29)	1.37 (0.89–2.11)	0.138
GG	6 (2)	4 (2)	1.04 (0.21–4.43)	1.000
A allele	509 (86)	316 (83)	0.79 (0.54–1.14)	0.197
G allele	81 (14)	64 (17)	1.27 (0.88–1.85)	0.197
LEPR Q223R				
TT	59 (20)	30 (16)	0.76 (0.45–1.26)	0.280
TC	140 (48)	93 (49)	1.08 (0.73–1.58)	0.709
CC	95 (32)	65 (35)	1.11 (0.74–1.66)	0.621
T allele	258 (44)	153 (40)	0.88 (0.67–1.15)	0.350
C allele	330 (56)	223 (59)	1.14 (0.87–1.50)	0.350
MC4R (rs17882313)				
CC	38 (13)	23 (12)	0.93 (0.51–1.67)	0.889
TC	127 (43)	76 (40)	0.88 (0.60–1.30)	0.511
TT	130 (44)	91 (48)	1.17 (0.80–1.71)	0.455
C allele	203 (34)	122 (32)	0.90 (0.68–1.20)	0.486
T allele	386 (66)	258 (68)	1.11 (0.84–1.48)	0.486
FTO (rs9939609)				
AA	32 (11)	21(11)	1.02 (0.54–1.90)	1.000
AT	125 (42)	84 (44)	1.08 (0.73–1.58)	0.708
TT	138 (47)	85 (45)	0.92 (0.63–1.35)	0.709
A allele	189 (32)	126 (33)	1.05 (0.79–1.40)	0.726
T allele	401 (68)	254 (67)	0.95 (0.72–1.26)	0.726

NCEP: National Cholesterol Education Program, LEPR: leptin receptor, OR: odd ratio, CI: confidence interval, MC4R: melanocortin-4-receptor, FTO: fat mass and obesity associated.

**Table 3.** Genotype And allele Frequencies Of Obesity-Associated Polymorphisms In Patients With And Without The Metabolic Syndrome As Determined By The IDF Definition

*Genotype/Allelotype*	*IDF (yes) n (%)*	*IDF (no) n (%)*	*OR (95% CI)*	p*-value*
Adiponectin 45 T→G				
TT	204 (70)	138 (71)	1.02 (0.67–1.55)	0.920
TG	79 (27)	52 (27)	0.97 (0.63–1.49)	0.889
GG	7 (2)	5 (3)	1.06 (0.26–3.96)	0.917
T allele	487 (84)	328 (84)	1.01 (0.70–1.46)	0.955
G allele	93 (16)	62 (16)	0.99 (0.69–1.42)	0.955
Adiponectin 276 G→T				
GG	185 (64)	109 (56)	0.72 (0.49–1.06)	0.081
GT	89 (31)	75 (38)	1.41 (0.95–2.10)	0.076
TT	16 (6)	11 (6)	1.02 (0.42–2.41)	0.954
G allele	459 (79)	293 (75)	0.80 (0.58–1.09)	0.142
T allele	121 (21)	97 (25)	1.26 (0.91–1.72)	0.142
LEPR K109R				
AA	211 (73)	139 (71)	0.93 (0.61–1.42)	0.722
AG	73 (25)	52 (27)	1.08 (0.70–1.67)	0.712
GG	6 (2)	4 (2)	0.99 (0.20–4.24)	0.989
A allele	495 (85)	330 (85)	0.94 (0.65–1.38)	0.755
G allele	85 (15)	60 (15)	1.06 (0.73–1.54)	0.755
LEPR Q223R				
TT	56 (19)	33 (17)	0.86 (0.52–1.41)	0.528
TC	140 (48)	93 (48)	0.99 (0.68–1.45)	0.956
CC	93 (32)	67 (35)	1.12 (0.75–1.68)	0.563
T allele	252 (44)	159 (41)	0.91 (0.69–1.19)	0.459
C allele	326 (56)	227 (58)	1.10 (0.84–1.45)	0.459
MC4R (rs17882313)				
CC	39 (13)	22 (11)	0.82 (0.45–1.47)	0.577
TC	119 (41)	84 (43)	1.09 (0.74–1.60)	0.707
TT	132 (46)	89 (46)	1.01 (0.69–1.47)	1.000
C allele	197 (34)	128 (33)	0.95 (0.72–1.26)	0.729
T allele	383 (66)	262 (67)	1.05 (0.80–1.40)	0.729
FTO (rs9939609)				
AA	22 (11)	21 (11)	0.97 (0.52–1.81)	1.000
AT	125 (43)	84 (43)	0.10 (0.68–1.47)	1.000
TT	133 (46)	90 (46)	1.01 (0.69–1.48)	1.000
A allele	189 (33)	126 (32)	0.99 (0.74–1.31)	0.944
T allele	391 (67)	264 (68)	1.01 (0.76–1.35)	0.944

IDF: International Diabetes Federation, LEPR: leptin receptor, OR: odd ratio, CI: confidence interval, MC4R313: melanocortin-4-receptor, FTO: fat mass and obesity associated.

The relationship between the obesity-associated polymorphisms studied here and the clinical and biochemical components of the metabolic syndrome was assessed. Significant associations are shown in Table 4. The TT genotype of the MC4R-associated polymorphism was found more frequently in patients with lower triglyceride levels (OR 1.55; 95% CI 1.05–2.28; *p* = 0.024), while the major TT genotype of the adiponectin 45T→G polymorphism occurred significantly more frequently in patients with normal fasting blood glucose levels (OR 1.92; 95% CI 1.21–3.09; *p* = 0.004). The TT genotype of the LEPR Q223R polymorphism was associated with low HDL cholesterol levels (OR 2.35; 95% CI 1.28–4.52; *p* = 0.003).

**Table 4.** Association Between Obesity-Related Polymorphisms And Individual Criteria Of The Metabolic Syndrome

*Genotype*	*Triglycerides < 1.7*	*Triglycerides ≥ 1.7*		
	*n (%)*	*n (%)*	*OR (95%CI)*	p*-value*
MC4R (rs17882313)				
CC	21 (12)	40 (13)	0.85 (0.46–1.55)	0.672
TC	65 (36)	135 (45)	0.69 (0.46–1.02)	0.056
TT	95 (53)	125 (42)	1.55 (0.105–2.28)	0.024*
	*Glucose < 5.6*	*Glucose ≥ 5.6*		
Adiponectin 45 T→G				
TT	128 (79)	212 (66)	1.92 (1.21–3.09)	0.004*
TG	31 (19)	99 (31)	0.53 (0.32–0.85)	0.006*
GG	3 (2)	9 (3)	0.65 (0.11–2.66)	0.740
	HDL (males < 1.03) (females < 1.29)	HDL (males > 1.03) (females > 1.29)		
LEPR Q223R				
TT	68 (22)	16 (11)	2.35 (1.28–4.52)	0.003*
TC	134 (44)	81 (55)	0.64 (0.42–0.97)	0.027
CC	101 (33)	49 (34)	0.99 (0.64–1.54)	1.000

*Implies significance at the 5% level of significance. Glucose: fasting blood glucose levels, MC4R: melanocortin-4-receptor, LEPR: leptin receptor, HDL: high-density lipoprotein.

A possible synergistic relationship between the six polymorphisms studied and the metabolic syndrome was analysed by counting the number of variant alleles carried by each individual. No relationship was observed between the cumulative polymorphic load and the metabolic syndrome by either definition, or the control subjects.

## Discussion

The metabolic syndrome is a common finding in young South African Indians with AMI, irrespective of the definitive criteria used (61% for NCEP ATP III and 60% for IDF). Male subjects were in the majority, but proportionally more females were found to have the metabolic syndrome (*p* = 0.007 and 0.02 for NCEP ATP III and IDF, respectively). These results are in agreement with previous studies, which reported a greater prevalence of the metabolic syndrome in women compared with men.^28^,^29^

Another interesting observation in our young patients was the increased prevalence of visceral obesity with the metabolic syndrome, as assessed by waist circumference measurements. Although only 19% of subjects in this study cohort were considered to be obese based on BMI measurements, it has been reported recently in South Asians that the risk level for the development of an adverse metabolic profile, with respect to body fat, is reached at a much lower BMI (21 kg/m^2^) compared to Europeans (≥ 30 kg/m^2^).^30^ Furthermore, the predominance of visceral adipose tissue with increasing waist circumference in Asian Indians^31^ has been shown to be associated with a higher prevalence of the metabolic syndrome, compared with African–Americans in whom subcutaneous fat predominates.^32^

Therefore the higher frequency of obesity and the metabolic syndrome in the South African Asian Indian patients in this study may explain in part the accelerated onset of atherosclerotic disease in these subjects compared to other ethnic groups. This concurs with other studies on CHD in Asian Indians, in whom about half of all myocardial infarctions occured in individuals under the age of 50, with 25% being under 40 years of age.^33^

With respect to the polymorphic variants in the adiponectin, LEPR, MC4R and FTO genes, no significant differences in allele frequency or genotype distribution were observed between patients with the metabolic syndrome, irrespective of the definition used, compared to those who did not have the syndrome. A previous study by Filippi *et al*.^34^ demonstrated a significant association between the adiponectin 276G→T polymorphism and the early onset of CHD. No similar relationship was found in our young patients with AMI for either the adiponectin 45T→G or 276G→T polymorphisms, which agrees with the findings of Jung *et al*. in their Korean subjects.^35^

Given the complexity of the metabolic syndrome and the lack of clarity surrounding its definition, most reports to date have been restricted to the examination of the relationship between variant polymorphisms and the individual criteria of the metabolic syndrome, rather than with the syndrome as a whole. For example, individuals carrying the adiponectin 276G→T polymorphism were found to be associated with type 2 diabetes mellitus in the Japanese population.^36^ In European subjects, conflicting results have been reported on the relationship between the LEPR Q223R polymorphism and obesity.^37^,^38^ Common polymorphisms in the MC4R and the FTO genes have, however, been shown to be predictive of obesity and diabetes.^39^

In the current study, the TT genotype of the adiponectin 45T→G gene occurred more frequently in patients with normal fasting blood glucose levels (*p* = 0.004), suggesting a protective influence of the major allele, while the TT genotype of the MC4R-associated polymorphism was found more frequently in patients with low triglyceride levels (*p* = 0.024). In contrast, the TT genotype of the LEPR Q223R polymorphism was strongly associated with low HDL cholesterol levels (*p* = 0.003). To the best of our knowledge, this is the first time that these findings have been reported in the Asian Indian population, and clearly warrant further evaluation in larger studies of different ethnic backgrounds. No other associations were found for any of the other polymorphic variants examined with any individual criteria of the NCEP ATP III and IDF definitions of the metabolic syndrome, including obesity.

Several limitations of this study merit consideration. Serum adiponectin and leptin levels were not measured, and therefore the functional significance of the polymorphisms studied cannot be assessed. Previous findings on the association between adiponectin polymorphisms and serum adiponectin levels have been contradictory,^40^,^41^ suggesting that serum levels may not necessarily reflect the overall amount of adiponectin in the body or its concentration in the interstitial space. With respect to subject numbers, the present study population was too small to achieve adequate statistical power in the case of some polymorphisms. However, the restriction of the study group to young patients drawn from a homogeneous population base limits the effects of non-genetic determinants, and provides an advantage in the evaluation of genetic polymorphic variation and associative comparisons.

## Conclusions

Our results show that the metabolic syndrome, as defined by both the NCEP ATP III and IDF criteria, is a common occurrence in young Asian Indian patients with AMI. Although obesity occurs frequently in these individuals, no significant association was found with any of the obesity-associated polymorphisms studied and the metabolic syndrome, or with obesity as determined by both waist circumference and BMI. This lack of association mostly likely reflects the complex pathogenesis of obesity, which involves environmental factors in addition to genetic components. Certain genotypes, most notably the TT genotype of the MC4R-associated gene and the TT genotype of the adiponectin 45T→G polymorphism may, however, exert a protective effect on individual components of the metabolic syndrome, such as blood glucose and triglyceride levels, while others such as the TT genotype of the LEPR Q223R gene was associated with adverse HDL cholesterol levels.
